# Assessment of efficacy and safety of dose-dense doxorubicin and cyclophosphamide (ddAC) in combination with immunotherapy in early-stage triple-negative breast cancer

**DOI:** 10.1007/s10549-024-07354-2

**Published:** 2024-05-21

**Authors:** Olivia White, Susan Dent, Kelly Westbrook, Hui-Jie Lee, Chengxin Yang, Heather N. Moore

**Affiliations:** 1 Department of Pharmacy, University of Virginia Health System, Charlottesville, VA USA; 2 Duke Cancer Institute, Durham, NC USA; 3 Department of Biostatistics and Bioinformatics, Duke University, Durham, NC USA

**Keywords:** Pembrolizumab dosing, Triple-negative breast cancer, Dose-dense

## Abstract

**Purpose:**

This study aimed to assess safety and efficacy of a modified KEYNOTE 522 protocol, which incorporated pembrolizumab every 6 weeks, allowing for concomitant dose-dense (14 day) doxorubicin and cyclophosphamide (ddAC). By optimizing this dosing, the intention of this modified protocol was to improve pathologic complete response (pCR) rates in a population associated with a poorer prognosis.

**Methods:**

This was a retrospective, single-center, cohort study. Patients were included if they had early stage, triple-negative breast cancer, and received at least one dose of AC. The entire cohort received neoadjuvant chemotherapy including weekly carboplatin and paclitaxel with pembrolizumab every 3 weeks for 12 weeks (4 cycles). The group then received either ddAC with pembrolizumab 400 mg every 6 weeks, or AC with pembrolizumab 200 mg every 3 weeks. The primary objective was pCR rate at time of surgery.

**Results:**

This study assessed outcomes in 25 patients over 34 months. The pCR rate in the pembrolizumab, AC 3-week cohort was 64.3% versus 81.8% in the ddAC and 6-week pembrolizumab group. No pembrolizumab-associated grade 3–4 adverse events occurred in the either cohort. Despite seeing an increased incidence of grade 3–4 toxicities in the ddAC arm, this did not result in additional chemotherapy delays or dose reductions.

**Conclusion:**

This study demonstrated tolerability and a potential for favorable outcomes with this patient population, making this modified KEYNOTE 522 protocol a reasonable treatment approach. Larger, prospective studies are warranted to assess the feasibility of this dosing and true optimization of patient outcomes given the small sample size of this study.

## Introduction

Triple-negative breast cancer (TNBC) is a heterogeneous and aggressive subgroup that accounts for approximately 10–20% of breast cancer diagnoses [1]. Administration of neoadjuvant chemotherapy (NAC) has demonstrated a survival benefit for those who achieve a pathologic complete response (pCR) following treatment [2]. The use of pCR is accepted by regulatory agencies as a surrogate marker for improved clinical outcomes. The highest rates of pCR following NAC administration are seen in triple-negative and human epidermal growth factor receptor-2 positive (HER2+) patients, thus making the use of NAC a preferred treatment option [2, 3]. The KEYNOTE 522 study specifically investigated the use of systemic chemotherapy, with or without pembrolizumab (given both in the neoadjuvant and adjuvant setting), for patients with stage II–III TNBC [4]. This study demonstrated a significant improvement in pCR rates, regardless of PD-L1 status, with 64.8% of patients achieving a pCR in the pembrolizumab arm versus 51.2% in the control arm. However, the implementation of the KEYNOTE 522 protocol in clinical practice has proven to be difficult. In the KEYNOTE 522 trial, pembrolizumab was administered with doxorubicin (A) or epirubicin, and cyclophosphamide (C), every 21 days to accommodate for the pembrolizumab dosing schedule. However, doxorubicin and cyclophosphamide are standardly given every two weeks in a dose-dense fashion (ddAC) instead of every 21 days due to the overall survival (OS) benefit reported with the dose-dense AC arm in CALGB 9741 [5].

In the KEYNOTE 555 study, administration of pembrolizumab every 6 weeks versus every 3 weeks was deemed safe and effective in patients with unresectable or metastatic melanoma [6]. Specifically, this study found maintenance of pharmacokinetic effects with pembrolizumab, despite the extended dosing interval, without compromising efficacy. Based on these results, the United States Food and Drug Administration (FDA) granted accelerated approval for pembrolizumab dosing every 6 weeks [7]. Following this update, more pharmacokinetic data has been published in support of this 6 week strategy; however, there is little data to date assessing this schedule in patients with breast cancer [8–11]. Our institution modified the KEYNOTE 522 protocol to employ ddAC with pembrolizumab every 6 weeks in hopes of improving clinical outcomes and consolidating systemic therapy.

Additionally, the use of adjuvant capecitabine for patients with residual disease (RD), as per the Create-X study, was not permitted in the KEYNOTE 522 protocol [12]. With Create-X demonstrating a five-year OS of 89.2% in the adjuvant capecitabine group versus 83.5% in the control group, patients at our institution with RD are treated concurrently with capecitabine and pembrolizumab following surgery. One study to-date has been published assessing the safety of concomitant nivolumab and capecitabine for TNBC patients; however, this cohort did not focus on the use of pembrolizumab and contained a limited sample size, warranting further investigation into the safety of these agents when used concomitantly [13].

The objective of this retrospective study is to determine the clinical efficacy and safety of this modified KEYNOTE 522 protocol at Duke Cancer Insitute (DCI) in patients with early stage TNBC scheduled to receive NAT.

## Methods

### Design and settings

**Fig. 1:**
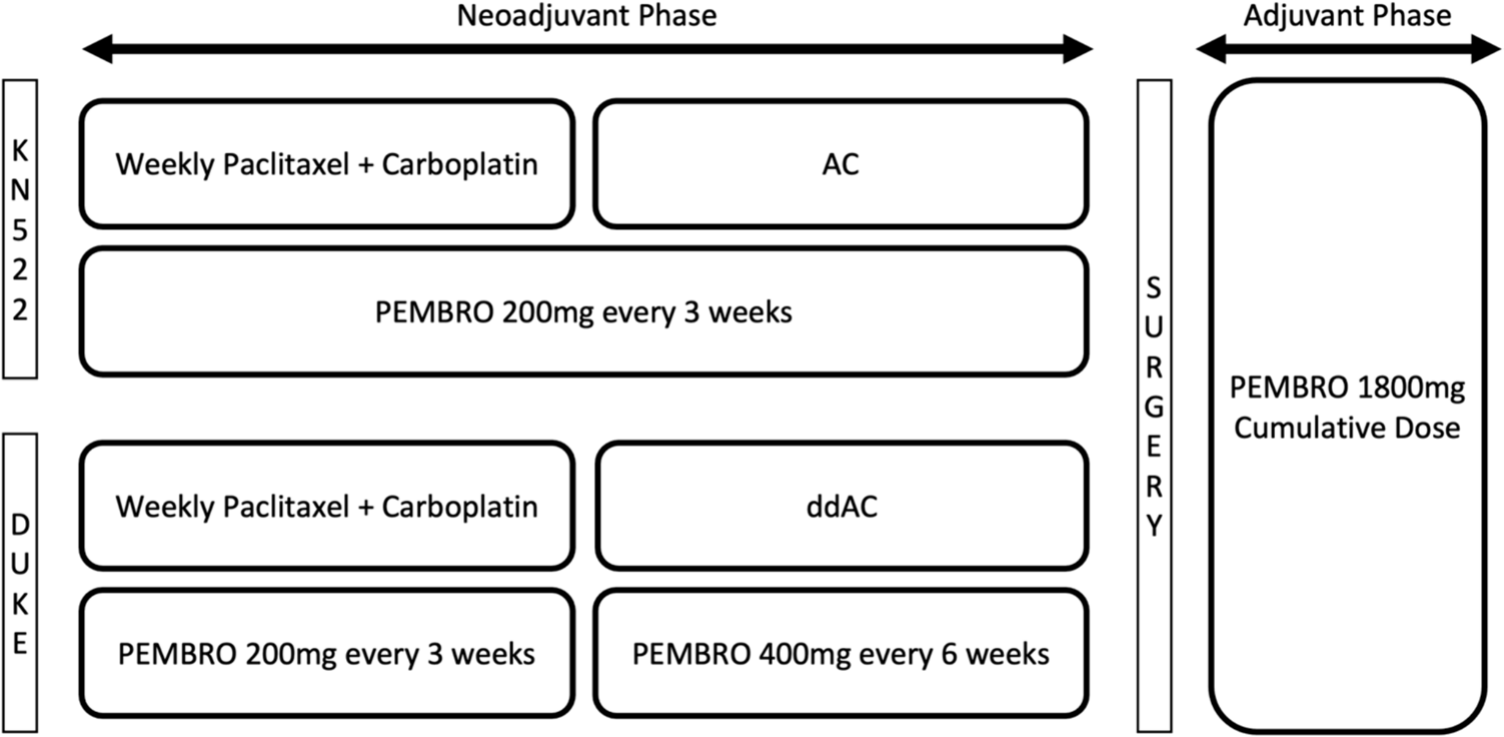
Duke KEYNOTE 522 modified protocol as compared to KEYNOTE 522

This single-center, retrospective, cohort study assessed the efficacy and safety of pembrolizumab 400 mg every 6 weeks in patients with TNBC that were initiated on NAC with ddAC, as demonstrated in Fig. 1. Patients received either AC with pembrolizumab 200 mg every 3 weeks based on the original KEYNOTE 522 protocol or, based on provider preference, patients received pembrolizumab 400 mg every 6 weeks with ddAC per the modified KEYNOTE 522 protocol. All patients received every 3 weeks AC or ddAC following completion of weekly carboplatin AUC 1.5 mg h/L plus paclitaxel 80 mg/m^2^ given with pembrolizumab 200 mg every 3 weeks for 12 weeks. Post-operatively, patients received a total of 1800 mg of pembrolizumab given as either 4 cycles of 400 mg with a 5th cycle of 200 mg, or pembrolizumab 200 mg for 9 cycles. The selected adjuvant pembrolizumab schedule was dependent on previous tolerance and patient preference. Patients were evaluated for the pre-specified outcomes for the duration of pembrolizumab therapy and up to 1 year following surgery.

Adult patients ≥ 18 years of age with a diagnosis of early-stage TNBC who received at least one dose of neoadjuvant AC and pembrolizumab at the Duke Cancer Institute (DCI) from February 1, 2020 to December 31, 2022, and who had undergone surgery during this timeframe were included. Patients were excluded if their systemic therapy was administered outside of the DCI, if they received this regimen in the adjuvant setting, or if given as part of a clinical trial.

### Data source and data collection

Data were collected for all patients including demographics, oncologic history, and clinical monitoring through a retrospective chart review. Data was managed using the Research Electronic Data Capture (REDCap) electronic data capture tools hosted at Duke University. REDCap is a secure, web-based software platform designed to support data capture for research studies. TNBC status was defined as less than or equal to 10% expression of hormone receptors (estrogen and progesterone) and HER2 negative on immunohistochemistry (IHC) or non-amplified on florescence in situ hybridization (FISH) on original pathology. Routine clinical and laboratory assessments, including safety outcomes and dose modifications, were assessed with every infusion.

### Measures

The primary outcome was the pCR rate with the implementation of ddAC plus pembrolizumab 400 mg every 6 weeks as compared to patients receiving pembrolizumab 200 mg and AC every 3 weeks. pCR was defined as the pathologic stage ypT0/Tis ypN0 at the time of surgical intervention as reported on surgical pathology. Secondary outcomes included the incidence of adverse events (AEs), dose modifications, and deferred cycles secondary to both immunotherapy and concomitant chemotherapy. AEs were graded according to CTCAE criteria version 5.0. Additional outcomes included the incidence of AEs, dose modifications, or deferred cycles with use of concomitant adjuvant capecitabine and pembrolizumab.

### Statistical analysis

Continuous variables were summarized with median and interquartile range (IQR). Categorical variables were summarized as frequency and percent. The primary outcome, pCR rate, was summarized by percentages with corresponding 95% exact binomial confidence intervals (CIs) and compared between treatment groups using Fisher’s exact test. Safety events and therapy modifications were summarized descriptively overall during the study period. All statistical analyses were performed in R 4.1.3 (R Core Team 2022) and the level of statistical significance was set at 0.05.

## Results

A total of 25 patients met inclusion criteria with 14 participants in the three-week AC and pembrolizumab 200 mg group, and 11 in the ddAC and pembrolizumab 400 mg group. Patient demographic and clinical characteristics are outlined in Table 1. The median (IQR) age of the cohort was 51 years (44–59). Notably, most patients had stage II disease at diagnosis. Furthermore, for patients who received pembrolizumab 400 mg every 6 weeks in the neoadjuvant setting, 27.3% returned to pembrolizumab 200 mg every 3 week dosing following surgery.

**Table 1:** Patient demographics and characteristics

	PEMBRO 200 mg/AC (n = 14)	PEMBRO 400 mg/ddAC (n = 11)	Total (n = 25)
Age, median (IQR)	50.5 (44.5–57.5)	52 (44–62)	51 (44–59)
Race, n (%)			
Caucasian	4 (28.6)	6 (54.5)	10 (40)
African American	9 (64.3)	3 (27.3)	12 (48)
Asian	1 (7.1)	1 (9.1)	2 (8)
Hispanic	0 (0)	1 (9.1)	1 (4)
Baseline performance status, n (%)			
0	13 (92.9)	10 (90.9)	23 (92)
1	1 (7.1)	1 (9.1)	2 (8)
Baseline menopausal status, n (%)			
Pre-menopausal	6 (42.9)	5 (45.5)	11 (44)
Post-menopausal	6 (42.9)	6 (54.5)	12 (48)
Neither	2 (14.3)	0 (0)	2 (8)
Stage at diagnosis, n (%)			
I	2 (14.3)	1 (9.1)	3 (12)
II	11 (78.6)	6 (54.5)	17 (68)
III	1 (7.1)	4 (36.4)	5 (20)
Adjuvant PEMBRO dose, n (%)			
No adjuvant PEMBRO	0 (0)	1 (9.1)	1 (4)
200 mg every 3 weeks	14 (100)	3 (27.3)	17 (68)
400 mg every 6 weeks	0 (0)	7 (63.6)	7 (28)

Table 2 summarizes the clinical efficacy and safety outcomes by treatment groups. pCR rates were 64.3% (9/14, 95% CI 48.2–97.7%) in the three-week AC and pembrolizumab 200 mg group versus 81.8% (9/11, 95% CI 35.1–87.2%) in the ddAC and pembrolizumab 400 mg group (p = 0.41). AEs secondary to chemotherapy occurred frequently across both cohorts. The most common grades 3–4 AEs included anemia, neutropenia, and infection of any kind. The rate of having at least one grade 3–4 AEs was 42.9% in the three-week AC arm and 72.7% in the ddAC arm. Despite seeing a higher incidence of grade 3–4 AEs in the ddAC arm, there were numerically lower rates of deferred cycles. The rate of having at least one deferred cycle across therapy was 80% overall, 85.7% in the three-week AC cohort, and 72.7% in the ddAC cohort. 75% (39/52) of all deferred cycles were due to an AE, with the most common AEs prompting deference being neutropenia (15/52, 28.8%), peripheral neuropathy (5/52, 9.6%), and infusion reactions (5/52, 9.6%). Dose modifications throughout therapy were more common in the ddAC arm with 81.8% of patients requiring at least one dose modification versus 71.4% in the standard dosing arm. Of all dose adjustments that occurred during the study period, 81.8% (36/44) were due to an ongoing AE, with the most common being peripheral neuropathy (9/44, 20.5%), neutropenia (8/44, 18.2%), and anemia (5/44, 11.4%).

**Table 2:** Outcomes by treatment group

	PEMBRO 200 mg/AC (n = 14)	PEMBRO 400 mg/ddAC (n = 11)	Total (n = 25)
pCR at time of surgery			
n (%)	9 (64.3)	9 (81.8)	18 (72.0)
95% CI	[48.2–97.7]	[35.1–87.2]	[50.6–87.9]
Adverse event and causal agent, n (%)			
Paclitaxel and carboplatin	14 (100)	11 (100)	25 (100)
Doxorubicin and cyclophosphamide	13 (92.9)	11 (100)	24 (96)
PEMBRO	11 (78.6)	4 (36.4)	15 (60)
Grade 3–4 adverse event and causal agent, n (%)	6 (42.9)	8 (72.7)	14 (56)
Paclitaxel and carboplatin	5 (35.7)	3 (27.3)	8 (32)
Doxorubicin and cyclophosphamide	4 (28.6)	7 (63.6)	11 (44)
Patients with at least one deferred cycle, n (%)	12 (85.7)	8 (72.7)	20 (80)
Patients with at least one dose adjustment, n (%)	10 (71.4)	9 (81.8)	19 (76)
Patients who discontinued treatment	8 (57.1)	1 (9.1)	9 (36)

Immunotherapy-related adverse events (irAEs) were seen in 60% of participants, with 78.6% of the patients receiving pembrolizumab 200 mg experiencing at least one irAE versus 36.4% of those receiving pembrolizumab 400 mg. One patient in the AC 3-week cohort had one cycle deferred due to ongoing thyroiditis; however, no other patients in either group experienced irAEs that led to deferred cycles or dose adjustments. No patients in either group experienced grade 3–4 irAEs; however, one patient did develop pembrolizumab-associated iritis. The most common AEs attributable to pembrolizumab included cutaneous reactions (10/25, 40%), thyroid disorders (6/25, 24%), and diarrhea (2/25, 8%). The median time of onset was 41 days and 91 days for cutaneous reactions and thyroid disorders, respectively. Cutaneous reactions occurred in 50% (7/14) of the patients in the neo-adjuvant pembrolizumab 200 mg cohort and 27.3% (3/11) of the patients in the neo-adjuvant pembrolizumab 400 mg cohort. Thyroid disorders occurred in three patients within each arm. The median cumulative dose of adjuvant pembrolizumab administered at the time of data cutoff was 1500 mg; however, 14 of the 25 patients remained on adjuvant pembrolizumab therapy at the end of the data collection period.

Five patients were administered concomitant adjuvant pembrolizumab and capecitabine. Of the five patients, three deferred cycles due to capecitabine-associated AEs. All but one of these AEs were CTCAE grade 1–2, with the one grade 3–4 AE being diarrhea. Due to the potential for both the pembrolizumab and capecitabine to contribute to this AE, both agents were discontinued at diarrhea onset. Two patients reported no AEs from either capecitabine or pembrolizumab.

## Discussion

There is limited literature addressing the safety and efficacy of a modified KEYNOTE 522 protocol with concurrent administration of ddAC and pembrolizumab 400 mg in a 6 week dosing interval. Our single-institution retrospective study demonstrated the feasibility and safety of a modified KEYNOTE 522 protocol.

In this cohort study, patients receiving the original KEYNOTE 522 regimen had a pCR rate of 64.3%, compared to a pCR rate of 81.1% in the modified KEYNOTE 522 regimen, suggesting a comparable benefit with the incorporation of dose-dense AC into the KEYNOTE protocol for early-stage TNBC. Larger, prospective, randomized studies are warranted to assess the true efficacy of ddAC when incorporated with the KEYNOTE 522 protocol and impact on pCR rates and overall outcomes compared to every three week AC.

In KEYNOTE 522, the most common overall AEs were febrile neutropenia, anemia, and pyrexia, with the most common grade 3–4 AEs being anemia, neutropenia, and elevated alanine aminotransferase. Treatment-related AEs led to discontinuation in 23.3% of patients. In this study, despite having a relatively high rate of cycles either deferred or doses modified, the discontinuation rate of ddAC with pembrolizumab 400 mg was 9.1%, suggesting that this regimen is tolerable for patients. Furthermore, the AEs that most commonly lead to treatment modifications or deference were similar to the original KEYNOTE 522 study, including both anemia and neutropenia. More peripheral neuropathy and infusion reactions were seen in our study compared to KEYNOTE 522.

While this study saw comparable AEs to KEYNOTE 522 from cytotoxic chemotherapy, irAEs described in this real-world analysis occurred at a much higher rate, with 60% of patients experiencing at least one irAE. Notably, in this analysis, the pembrolizumab 200 mg cohort experienced almost double the rate of irAEs compared to the 400 mg group. Despite pharmacokinetic analyses supporting the 400 mg dosing interval, there is currently a lack of safety data comparing the two strategies, and no studies to-date assessing this dosing schema in breast cancer. As supported by the KEYNOTE 555 study, the C_max_ of the 400 mg dose is higher than the 200 mg dosing. However, it is still unclear how this increase in initial exposure impacts safety. One study assessed the safety of this dosing interval in advanced non-small cell lung cancer, and indeed found that there was an increased incidence of toxicity with the 400 mg cohort [9]. However, alternative studies have found no difference in time to treatment discontinuation nor any difference in overall survival [10, 11]. Therefore, based on the findings of our study and current literature, it is reasonable to discern that pembrolizumab 400 mg every 6 weeks is an administration schedule that can be considered not only pharmacokinetically effective, but also safe.

For the patients in this analysis, it is imperative to note the quantity of endocrine-specific irAEs seen in comparison to the literature. In the KEYNOTE 522 study, 13.7% of patients developed hypothyroidism, 4.6% of patients developed hyperthyroidism, and 2.3% developed adrenal insufficiency. The results seen in our study were fairly comparable; however, per the National Comprehensive Cancer Network, it is estimated that immunotherapy-induced hypothyroidism occurs at an incidence of approximately 3.8% in all patients who receive immunotherapy. It has been estimated that for patients with breast cancer, hypothyroidism and other endocrine-related irAEs occur at a higher rate, with this hypothesis being supported by both the KEYNOTE 522 study and our results. The reasoning for this remains unclear. However, it is well understood that women are generally more susceptible to autoimmune diseases, potentially due to a lack of protective effects from testicular hormones, fluctuating levels of ovarian hormones, and effects associated with sex chromosomes [14]. Furthermore, risk factors for irAEs have been proposed including age (< 60 years), concomitant immunotherapy and chemotherapy administration, and previous anthracycline exposure [15]. This may explain the increased incidence seen in the breast cancer population. Our study highlights the importance of monitoring for irAEs in women with TNBC treated in the real-world setting, especially those associated with endocrinopathies. Furthermore, these results emphasize the need for practitioners to be mindful of differential diagnoses for patients receiving immunotherapy, and to understand the true variation in presentation that can exist for patients who experience an irAE. Multidisciplinary approaches to these toxicities, including the implementation of irAE multidisciplinary teams, should be considered at institutions where this resource is available.

There are several limitations of our study. This was a small retrospective study focused on a single institution. Given the limited size of our cohort, data may not be representative of the true TNBC population and allowing for biases in the data collection process. Additionally, this limited sample size leads to the potential for underpowering our assessment, with the true impact on the differences in these outcomes needing to be further highlighted with larger studies. This study additionally is limited through the patients represented in our population. All involved patients received four cycles of doxorubicin and cyclophosphamide, naturally allowing for a proclivity to exclude those with more prominent toxicities, prompting therapy discontinuation either from chemotherapy or immunotherapy. Furthermore, the authors did not account for the quantity of neoadjuvant carboplatin administered to the patients, potentially confounding the results of this study. Lastly, this study utilized the CTCAE Version 5.0 to grade adverse events, whereas KEYNOTE 522 utilized the 4.0 grading system. While this difference does not have a clear clinical impact in the outcomes from our study, the authors felt this difference should be highlighted due to potential differences in AE reporting.

Given the results of our study, it is reasonable to consider modification of the KEYNOTE 522 protocol to administer ddAC with pembrolizumab 400 mg every 6 weeks. Our study also demonstrated that irAEs secondary to pembrolizumab are higher in a real-world population, thus warranting vigilant AE monitoring during therapy. Additional data is needed to further determine which patients derive the most benefit from immunotherapy, benefit from continued adjuvant pembrolizumab, and benefit from concomitant pembrolizumab and oral agents (eg. capecitabine or olaparib). Ongoing trials concentrated on these needs include those highlighting safety and efficacy of pembrolizumab these with oral agents (NCT04191135, NCT04683679, NCT05203445), antibody–drug conjugates (NCT05633654, NCT05675579), and the assessment of pembrolizumab completion in the setting of pCR rates (NCT03036488). Until further data is avaliable, the modified KEYNOTE 522 regimen in this study appears to be a safe and effective option in which clinicians can potentially optimize outcomes for a patient population that otherwise has a poorer prognosis. Future prospective data involving a larger group of patients is warranted to determine the true benefit of this dosing strategy.

## Data Availability

The datasets generated during and/or analyzed during the current study are not publicly available due to containing personal health information but are available from the corresponding author on reasonable request.
